# Biogenic *Punica granatum* Flower Extract Assisted ZnFe_2_O_4_ and ZnFe_2_O_4_-Cu Composites for Excellent Photocatalytic Degradation of RhB Dye

**DOI:** 10.3390/toxics12010077

**Published:** 2024-01-16

**Authors:** Amal Alshehri, Laila Alharbi, Aiyaz Ahmad Wani, Maqsood Ahmad Malik

**Affiliations:** 1 Chemistry Department, Faculty of Science, King Abdulaziz University, P.O. Box 80203, Jeddah 21589, Saudi Arabia; aal.shehri@bu.edu.sa (A.A.); lalhrbi@kau.edu.sa (L.A.); 2 Chemistry Department, Faculty of Sciences and Arts in Baljurashi, Albaha University, Albaha 65779, Saudi Arabia; 3 Department of Chemistry, Faculty of Sciences, Jamia Millia Islamia, New Delhi 110025, India; aiyazwani2020@gmail.com

**Keywords:** zinc ferrite, nanocomposites, photocatalysis, visible light, photodegradation

## Abstract

Globally, the textile industry contributes to pollution through accidental discharges or discharge of contaminated wastewater into waterways, significantly affecting water quality. These pollutants, including dye molecules, are environmental hazards for aquatic and terrestrial life. The field of visible light-mediated photocatalysis has experienced rapid growth, driven by the utilization of photocatalysts that can absorb low-energy visible light and effectively degrade dyes. In the present study, we report a simple method to controllably synthesize Fe_2_O_3_, ZnO, and ZnFe_2_O_4_ using the one-pot synthesis method. In the subsequent step, copper (Cu) was deposited on the surface of ZnFe_2_O_4_ (forming ZnFe_2_O_4_-Cu) using a facile, green, and cost-effective method. The synthesized samples were characterized using various techniques, including XRD, UV-Vis DRS, FT-IR, SEM-EDX, HR-TEM, XPS, PL, and BET analysis. These techniques were employed to investigate the composition, morphology, structure, and photophysical properties of as-prepared samples. The ZnFe_2_O_4_-Cu nanocomposite demonstrated efficient photocatalytic activity for degrading RhB dye pollutants under visible light. The photocatalyst was successfully reused for three consecutive cycles without significantly decreasing performance. Furthermore, during the study, the radical scavenging test emphasized the role of different radicals in the degradation of dye pollutants. This research has the potential to enable the efficient production of high-performance photocatalysts that can rapidly eliminate ecologically harmful dyes from aqueous solutions.

## 1. Introduction

Water is a critical resource for the existence of life on Earth and human survival. With increasing economic growth, there is an increase in the level of undesired pollution, such as organic dyes, heavy metals [1], and pharmaceuticals [2] in water bodies, which endanger people’s health and the ecosystem. Photocatalysts, the substrate that absorbs light and acts as catalysts, are widely used in organic dye degradation. They are characterized by low toxicity, low cost, and high stability [3]. Photocatalysis has many limitations, like stability and reusability of catalyst, recombination between hole and electron, and band gap [4]. Metal oxide nanoparticles MONPs are significantly attractive in dye degradation as a photocatalyst due to their flexible properties [5,6,7,8]. MONPs can be classified into different types based on the number of metals: monometallic (which contains only one metal), bimetallic (which contains two types of metals), and trimetallic (which contains three types of metals) [9,10]. Trimetallic NPs have attracted attention, where it was found that they have a promising synergistic effect of catalytic activity, which outweighs the properties of mono and bi-metallic NPs acting as antimicrobials [11,12,13,14,15]. For example, AuFeAg hybrid NPs and FeAgPt alloy [16] exhibited better catalytic activity when compared to mono and bi-metallic NPs [17,18]. MONPs can be synthesized through three approaches: physically (consuming a lot of energy and producing waste), chemically (which requires a lot of hazardous chemicals), and greenly (which uses biomolecules to minimize the toxicity of the MONPs process) [17,18,19]. Green synthesis of MONPs has recently gained popularity due to their environmental advantages and affordable cost [20,21,22,23]. The biomolecules, including bio-polymers [24], ascorbic acid [25], citric acid [15], flavonoids [26], nucleic acids, and proteins [27] derived from natural sources such as plant parts, fungi, and bacteria, play important roles in the capping and reduction of NPs [28]. Among them, plants are considered the most simple, facile, and cost-effective bio-sources, and they can work as a capping/stabilizing and reducing agent [29]. In order to reduce photogenerated electron and hole recombination and increase the range of light absorption, MONPs can be doped with metallic or non-metallic ions [30]. The different types of dopants have different effects on interactions with electrons and/or holes because of their different locations within the host lattice [30,31].

In general, photocatalysts should be cost-effective, non-toxic, and activated by visible/solar light properties [32]. Among other MONPs, ZnO, CuO, and Fe_2_O_3_ have attracted interest because of their versatile applicability and physiochemical characteristics. ZnO is an n-type semiconductor with fantastic properties such as safety, abundant natural availability, high surface area, high catalytic activity, and high stability against photo-corrosion [32,33,34]. However, it has a band gap of around 3.4 eV, which means it is not efficient in visible light [32,35,36]. Fe_2_O_3_ NPs are the most stable form of iron oxide and possess desirable characteristics such as excellent corrosion resistance, cost-effectiveness, biocompatibility, environmental friendliness, and non-toxicity [37]. Additionally, when doped onto ZnO NPs, Fe_2_O_3_ can reduce electron recombination and shift the absorbance to a higher wavelength [38]. Zinc ferrite (ZnFe_2_O_4_) is an iron-based semiconducting oxide that has a spinal structure and received significant interest because of its excellent magnetic properties with a low band gap of around 1.9 eV, which makes it a promising material to use as a photocatalyst [39]. However, it suffers from some drawbacks, such as a high rate of agglomeration and rapid recombination of the electron-hole pairs [39]. One of the most effective strategies to overcome these drawbacks is depositing by metallic NPs. Depositing copper (Cu) nanoparticles holds significant interest as a non-toxic additive material. Additionally, it is more cost-effective compared to noble metals. It serves as a trap for charge carriers, interfering with interfacial transfer and reducing electron-hole recombination [40,41,42]. ZnFe_2_O_4_ NPs are typically prepared using various methods, including sol-gel [43], co-precipitation [6], hydrothermal [44], microwave [45], microemulsion techniques [46], and biosynthesis [47]. These different methods offer flexibility in tailoring the properties of synthesized materials ZnFe_2_O_4_ NPs.

The pomegranate plant (*Punica granatum* L.) is abundantly available in the Middle East, and its cultivation has spread to various regions across the world [48]. *Punica granatum* plant extract possesses many metabolites, such as organic acids, sugars, flavonoids, polyphenols, fatty acids, anthocyanins, and vitamins [49], which can work as capping and reducing agents in the preparation of ZnFe_2_O_4_ NPs. Ascorbic acid is also a cost-effective and eco-friendly mild reducing agent and can also work as a capping/stabilizing agent [50]. Some research used sodium dodecyl sulfate (SDS) as a capping agent to improve NPs properties [51,52]. This work aims to prepare a low-toxic and cost-effective semiconductive photocatalysis of ZnO, Fe_2_O_3_, and ZnFe_2_O_4_ by the co-precipitation method by using *Punica granatum* extract as a reducing and capping agent instead of using toxic chemicals. After that, ZnFe_2_O_4_ will be deposited with Cu to enhance the dye degradation’s photocatalytic activity.

## 2. Experimental

### 2.1. Materials

*Punica granatum* flowers were purchased from the Saudi market in Jeddah. Metal precursor salts of Iron (II) chloride tetrahydrate (FeCl_2_⋅4H_2_O, purity 99.0%), iron (III) chloride hexahydrate (FeCl_3_⋅ 6H_2_O, purity 99.0%), zinc acetate-2-hydrate (C_4_H_6_O_4_Zn⋅2H_2_O, purity 99.5%), and copper(II) nitrate trihydrate (Cu(NO_3_)_2_⋅3H_2_O, purity > 99.5%) were obtained from Sigma-Aldrich, MO, USA. Other reagents, sodium dodecyl sulfate (C_12_H_25_OSO_3_Na, purity 99%), sodium hydroxide (NaOH, pellets), ascorbic acid (C_6_H_8_O_6_, purity 99%), and Rhodamine B dye (C_28_H_31_ClN_2_O_3_, purity ≥ 95%) were also purchased from Sigma-Aldrich USA. The chemical reagents were utilized in their original state without undergoing any additional purifying processes. The preparation of all solutions was done using deionized water.

### 2.2. Preparation of Fe_2_O_3_, ZnO, ZnFe_2_O_4_ and ZnFe_2_O_4_-Cu

In order to prepare the extract of *Punica granatum* flowers, the flowers underwent a series of washes using distilled water, with an additional wash using deionized water to remove any contaminants. The flowers were then dried and finely powdered. Following this, 10 g of the powder was added to 200 mL of deionized water. The mixture was then heated at 80 °C for two hours while being stirred magnetically. Subsequently, it was allowed to rest overnight before undergoing filtration and immediate utilization in the synthesis of nanoparticles.

The preparation of Fe_2_O_3_ and ZnO nanoparticles was carried out by a green co-precipitation method. Initially, equimolar aqueous solutions of FeCl_2_ (2M, 25 mL) and FeCl_3_ (2M, 25 mL) (1:1 ratio) were mixed under constant stirring. To this reaction mixture, 50 mL of freshly prepared aqueous extract of *Punica granatum* was added and stirred for 30 min at 70 °C, followed by the dropwise addition of 2 molar NaOH solution until the pH reached between 11 and 12. The reaction mixture turned to a dark brown precipitate. Similarly, 50 mL of 2M zinc acetate was mixed with 50 mL of freshly prepared aqueous extract of *Punica granatum.* In this reaction mixture, NaOH (2M) solution was added dropwise under vigorous stirring at 60 °C until the pH reached 12, and the light yellowish precipitate formation was observed. The precipitate of both reactions was collected by centrifugation and washed several times with deionized water and ethanol until pH 7 was attained. The acquired materials were collected and dried at 80 °C for 24 h, followed by the calcination at 500 °C for 4 h. For the preparation of ZnFe_2_O_4_, FeCl_2_ (2M, 25 mL) and FeCl_3_ (2M, 25 mL) (1:1 ratio) were mixed with 25 mL of 2M zinc acetate solution under vigorous stirring at 70 °C. To this reaction mixture, 100 mL of *Punica granatum* aqueous extract was added and stirred continuously for 30 min. A 2M NaOH solution was added to this reaction mixture to attain a pH of 12, and a deep brown precipitate was formed immediately. The precipitate was centrifuged, and the acquired solid material was washed multiple times with distilled water, followed by ethanol until the pH reached 7. The as-prepared samples were dried at 80 °C for 24 h, followed by the calcination for 4 h at 500 °C.

**Figure 1:**
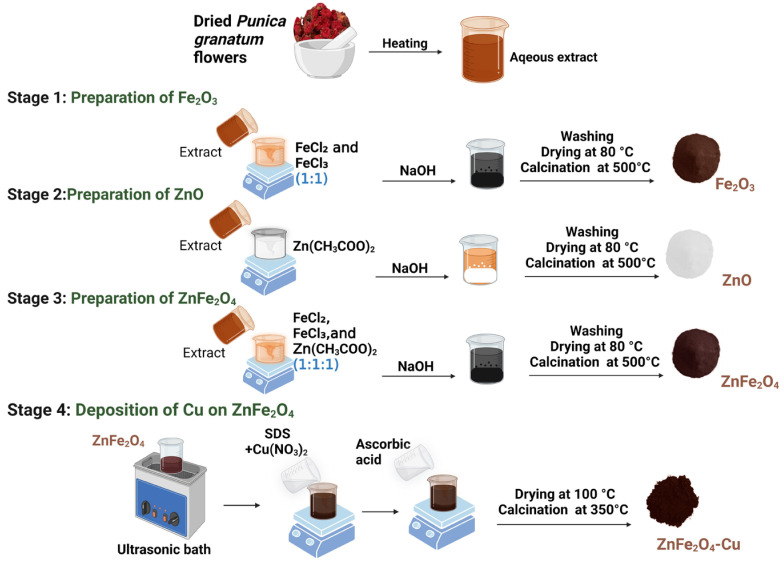
Schematic representation of the synthesis of Fe_3_O_4_, ZnO, ZnFe_2_O_4_, and ZnFe_2_O_4_-Cu.

ZnFe_2_O_4_-Cu nanoparticles were synthesized by dispersing 1 g of ZnFe_2_O_4_ NPs in 50 mL of deionized water using an ultrasonic bath for 10 min before being stirred. Then 5 mL of 0.1 M SDS and 10 mL of 0.1 M Cu(NO_3_)_2_ solution were added to dispersed ZnFe_2_O_4_ NPs suspension under continuous vigorous stirring. Then, 10 mL of 0.1 M ascorbic acid was added to complete the reduction process of Cu on the surface of the ZnFe_2_O_4_ NPs. After that, samples were dried and then calcined for three hours at 350 °C after being rinsed three times with distilled water and once with 99.9% ethanol. The complete synthesis of Fe_2_O_3_, ZnO, ZnFe_2_O_4_, and ZnFe_2_O_4_-Cu NPs is schematically shown in Figure 1.

### 2.3. Characterization

The as-prepared nanomaterials were characterized using spectroscopic and microscopic techniques to determine their surface morphology, purity, and physicochemical properties. In order to confirm the NPs structure, XRD patterns were collected using an X-ray diffractometer (D8, Advance, Bruker, Germany), utilizing Cu Kα radiation (λ = 1.5406 Å) over a range 5–80 2θ degrees. A Debye-Scherer equation was used to calculate the average crystallite size [53,54].

(1) $$D=\frac{K\lambda }{\beta \;Cos\theta }$$

Here, *D* = crystallites size (nm), *K* = shape factor, *λ* = wavelength of the X-ray sources, *β* = FWHM (radians), and *θ* = Peak position (radians). FT-IR was carried out for as-prepared NPs to determine the function group on the surface of NPs and to confirm the presence of M-O bonds. FT-IR was carried out by using FTIR spectrophotometer (Perkin Elmer, Waltham, MA, USA) using KBr in the range of 4000–400 cm^−1^. The UV-Vis diffused reflectance spectroscopy (UV-Vis DRS) spectra were conducted using a spectrophotometer manufactured by Shimadzu in Kyoto, Japan, to determine the band gap from the absorbance spectrum by using Tauc’s equation [55].

(*αhν*)^*γ*^ = A (*hν* − *Eg*) (2)

Here, *α* is the absorption coefficient, *h* is the Plank’s constant, *ν* is the frequency of photons, *A* is a proportionality constant, *γ* denotes the nature of the electronic transition, and *Eg* is a band gap energy. The X-ray photoelectron spectra (XPS) were measured using an X-ray photoelectron spectrometer model k Alpha manufactured by (Thermofisher, Waltham, MA, USA) with LAB 6 ion source to determine the coordination number of elements. Surface morphology and elemental compositions of as-prepared NPs were examined using the energy dispersive spectroscope (EDS) manufactured by Bruker, Berlin, Germany, coupled to the scanning electron microscope (FESEM model Nova Nano SEM 350 manufactured by FEI, Eindhoven, The Netherlands). The specimen holder utilized was made from aluminum. The sizes and shapes of the particles were determined using a transmission electron microscope (HR-TEM) model Technai 200 manufactured by FEI, Pleasanton, CA, USA. Photoluminescence spectra (PL) were obtained utilizing a fluorescence spectrophotometer manufactured by Perkin Elmer. Surface area, pore size, and pore volume, which are crucial properties of heterogeneous catalysts, were determined utilizing N_2_ adsorption-desorption using a Quantachrome Instrument v11.05 manufacturing by Anton Paar, Boynton Beach, FL, USA, Boynton Beach (USA) and Quantachrome Novawin ©1994–2018 software.

### 2.4. Photocatalytic Activity of ZnFe_2_O_4_-Cu

The photocatalytic reactivity of the as-prepared ZnFe_2_O_4_-Cu to visible light at room temperature was measured using the hetero-polyaromatic rhodamine B (RhB) dye as a probe molecule. Photodegradation of RhB dye was carried out using distilled water for the preparation of stock solution (100 mg/L) until completely dissolved, then kept at room temperature in a dark place. Every solution used in this study was prepared by diluting the stock solution of RhB dye. A UV-Vis spectrophotometer manufacturing by Thermo-Scientific evolution, Waltham, MA, USA, was used to measure the concentration of RhB dye. The suspension was then exposed to light from a 300 W Hg lamp with a cutoff filter (420 nm), with the lamp placed roughly 10 cm from the beaker. In the first, the photocatalytic degradation of RhB dye without ZnFe_2_O_4_-Cu was studied. After that, to test the performance of ZnFe_2_O_4_-Cu on RhB degradation, 2.0 g/L of ZnFe_2_O_4_-Cu NPs was added into 10 mL of 20 mg L^−1^ RhB solution. An aqueous suspension of RhB and ZnFe_2_O_4_-Cu photocatalyst was constantly agitated in the dark for 30 min to achieve adsorption-desorption equilibrium before being exposed to light. At intervals of specific time, 5 mL of the suspension was collected and centrifuged for 20 min at 5000 rpm. The initial pH of the samples was controlled by adding dilute solutions of HCl (1 M) and NaOH (1 M), and the pH was measured using a pH meter. The UV-visible absorption spectra of the supernatant were subsequently recorded using a Thermo-Scientific evolution UV-visible spectrophotometer.

The percentage of RhB dye degradation was calculated using the formula below.

(3) $$\%\;Dye\;degradation=\frac{C_{o}-C_{t}\;}{C_{o}}\times 100$$

Here, *C_o_* is the initial absorbance of RhB dye solution at *t* = 0, and *C_t_* is the absorbance at irradiation time ‘*t*’.

Different parameters, such as catalyst dosage, RhB dye concentration, and the effect of pH on dye degradation, were studied to optimize the photocatalytic efficiency of ZnFe_2_O_4_-Cu NPs. Additionally, the reusability of the ZnFe_2_O_4_-Cu NPs was investigated at optimum experimental conditions, which consisted of 2.0 g/L of ZnFe_2_O_4_-Cu nanocomposite and 20 mg/L RhB dye aqueous solution at pH 10. The recycling test of ZnFe_2_O_4_-Cu for RhB dye degradation was done by separation of photocatalyst after every cycle of exposure by centrifuge to retrieve the catalyst. After that, the collected catalyst was washed using absolute alcohol, followed by deionized water. The catalyst was then left to dry overnight at a temperature of 70 °C in the oven. After that, the collected nanocatalyst was introduced into a freshly prepared RhB dye reaction solution, initiating the subsequent cycle of the experiment.

The most active species responsible for the photocatalytic degradation of RhB dye using ZnFe_2_O_4_-Cu were investigated under optimal experimental conditions. This was accomplished by introducing benzoquinone (BQ) (1.0 mM) to quench (O_2_^•−^), isopropyl alcohol (IPA) (1.0 mM) to quench (^•^OH), and Ammonium oxalate (AO) (1.0 mM) to quench (h^+^).

## 3. Results and Discussions

### 3.1. X-ray Diffraction Analysis

**Figure 2:**
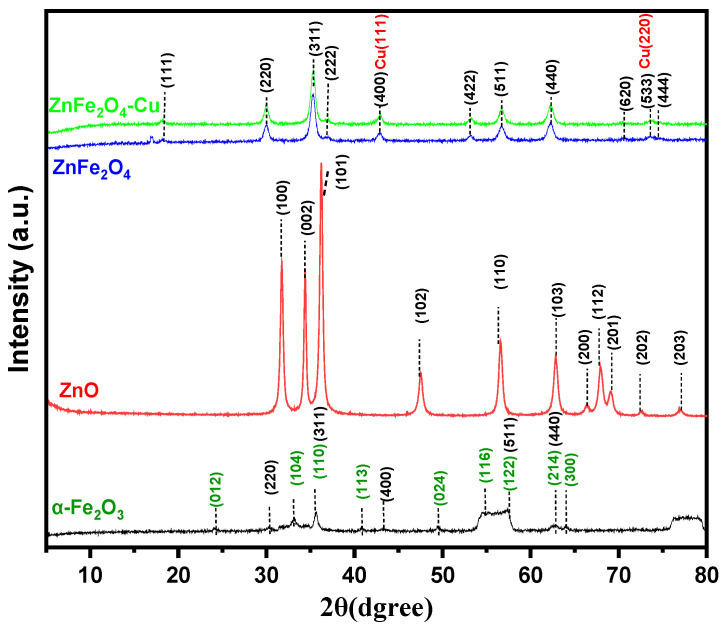
XRD patterns of Fe_2_O_3_, ZnO, ZnFe_2_O_4_ and ZnFe_2_O_4_-Cu NPs.

The phase purities, structure, and crystallite size of all samples were assessed using X-ray powder diffraction (XRD) analysis. XRD patterns were obtained by scanning at 2θ angles ranging from 10° to 80°, with a scan rate of 1°/per minute. Figure 2 shows the XRD patterns of as-prepared Fe_2_O_3_, ZnO, ZnFe_2_O_4_, and ZnFe_2_O_4_-Cu nanomaterials. The major XRD patterns of Fe_2_O_3_ exhibited the characteristic hematite (ICDD card no. 33-0664) pattern at 2θ = 24.40°, 33.39°, 35.83°, 40.99°,49.81°, 54.54°, 57.58°, 62.90°, and 64.11° that are related to Miller indices (012), (104), (110), (113), (024), (116), (122), (214), and (300) planes respectively and it is possible to index the observed peaks in accordance with the expected rhombohedral structure of α-Fe_2_O_3_ [56,57,58,59]. The other peaks are related to cubic magnetite NPs magnetite (Fe_3_O_4_). The X-ray diffraction pattern of ZnO nanoparticles exhibited characteristic peaks at specific 2θ values, namely 31.65°, 34.39°, 36.32°, 47.56°, 56.52°, 62.84°, 66.35°, 67.92°, 69.07°, 72.59°, and 76.97°. These peaks correspond to the crystallographic planes (100), (002), (101), (102), (110), (103), (200), (112), (201), (004), and (203), respectively. The observed peak positions are in good agreement with the reference JCPDS, File No. 036-1451 [60]. The XRD pattern of ZnFe_2_O_4_ nanoparticles recorded at room temperature in the 2θ ranges from 10–80° shown in Figure 2. The synthesized nanoparticles exhibited diffraction peaks at specific 2θ values, namely 18.23°, 30.0°, 35.35°, 36.75°, 42.73°, 53.08°, 56.70°, 62.23°, 70.56°, 73.55°, and 74.51°. These peaks corresponded to the (111), (220), (311), (222), (400), (422), (511), (440), (620), (533), and (444) Miller index planes of the synthesized zinc ferrite (ZnFe_2_O_4_) nanomaterials. This confirmed the cubic structure of ZnFe_2_O_4_ nanoparticles according to the JCPDS card No. 82-1042 [61]. There is not any impurity peak observed in the XRD pattern of ZnFe_2_O_4_, which indicates the purity and the high crystallinity of the prepared sample. During the synthesis of ZnFe_2_O_4_ nanoparticles, a decrease in the peak intensity (101) associated with ZnO was observed upon the introduction of Fe^3+^ ions, indicating a partial quenching effect. This phenomenon can be elucidated by the creation of ZnFe_2_O_4_, where the absence of Fe^3+^ incorporation results in a shift of signals towards higher angles. This shift can be attributed to the larger ionic radius of Zn^2+^ (0.074 nm) compared to that of Fe^3+^ (0.064 nm) [62]. Furthermore, it is evident that the two peaks at 42.73° and 73.55° overlap with the (111) and (220) crystallographic planes of copper (Cu). The observed peaks in the X-ray diffraction (XRD) pattern can be attributed also to a face-centered cubic (FCC) crystal structure of copper (Cu), as shown by the JCPDS reference number 71-4610 [63]. The Debye-Scherrer equation was used to determine the crystallite sizes of various nanoparticles. The computed sizes were found to be 14.90 nm for Fe_2_O_3_, 14.95 nm for ZnO, 9.00 nm for ZnFe_2_O_4_, and 9.57 nm for ZnFe_2_O_4_-Cu NPs. The observed alterations in the crystal phases can be elucidated as follows: upon heating to a temperature of 350 °C, the ZnFe_2_O_4_-Cu constituents underwent decomposition, resulting in the production of carbon dioxide and water. Additionally, the Fe, Zn, and Cu metal constituents reacted with the oxygen present in the surrounding atmosphere, leading to the formation of the ZnFe_2_O_4_-Cu nanocomposite. Additionally, despite these chemical changes, the nanocomposites morphology, particularly, the high specific surface area. Furthermore, in the samples pyrolyzed at 350 °C, the small ZnFe_2_O_4_-Cu nanocomposite particles gradually grew and became more crystalline [64,65].

### 3.2. FT-IR Analysis

**Figure 3:**
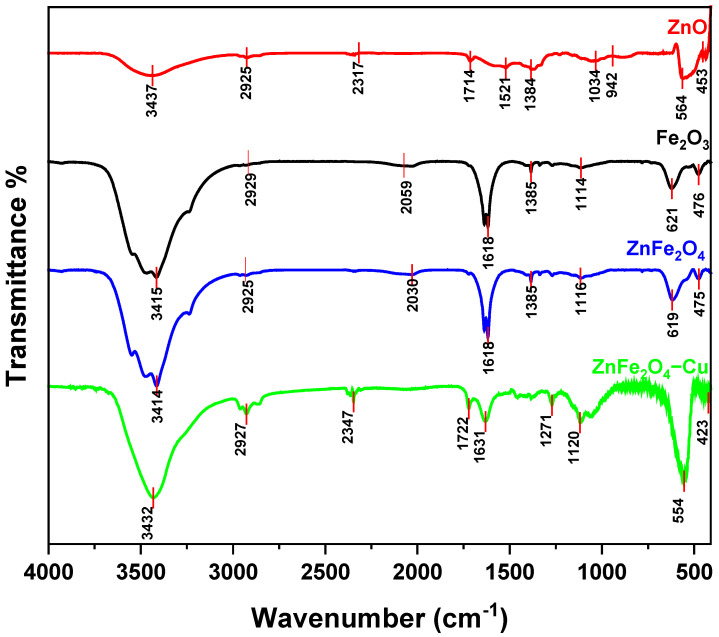
FT-IR analysis of Fe_2_O_3_, ZnO, ZnFe_2_O_4_, and ZnFe_2_O_4_-Cu NPs.

The formation of nanomaterials and metal–oxygen bond formation was further confirmed by functional group analysis, FT-IR spectroscopy. The FT-IR spectra shown in Figure 3 were recorded for the prepared samples in the range of 4000 and 400 cm^−1^, and the vibrational signals were analyzed. A broad band at 3100–3700 cm^−1^ can be returned to the stretching and bending vibrations of the hydroxyl groups or the surface-adsorbed water molecules [66]. The sharp absorption band at around 1618 and 1385 cm^−1^ could assigned to C=O stretching frequency and bending vibrations of the hydroxide (OH) group of water molecules absorbed at the surface of samples [67]. On the other hand, the peaks at 1114 cm^−1^ and 2929 cm^−1^ are attributed to the presence of C–O and C–H vibration modes [68]. These observed peaks are related to different phytochemicals present in the *Punica granatum* flower extract, which act as reducing and stabilizing agents in the solution to prevent nanoparticle aggregation. These results agree with the previously reported literature [69,70]. Subsequently, the observation of two distinct spectral bands with frequencies of 621 cm^−1^ and 476 cm^−1^ was made, which can be ascribed to the vibrational mode of Fe–O within the rhombohedral crystal structure of hematite (Fe_2_O_3_), as reported in a previous study [71]. The stretching vibrations of the Zn–O bond in the tetrahedral site of ZnO can be attributed to the two absorption bands observed at 564 cm^−1^ and 453 cm^−1^ [72]. The primary source of metal−O stretching is the variations in distances that lead to Fe^3+^−O^2−^ when the metal ion occupies both octahedral and tetrahedral sites. The Zn–O bond (tetrahedral Zn ion) exhibits a characteristic vibrational peak at 619 cm^−1^, whereas Fe−O in octahedral configuration exhibits another peak at 475 cm^−1^. The current findings are supported by the observation of two characteristic bands, one at a lower frequency peak (450 cm^−1^–400 cm^−1^) indicating the octahedral sites and one at a higher frequency (600 cm^−1^–550 cm^−1^) corresponding to tetrahedral sites [73,74,75]. ZnFe_2_O_4_-Cu composite shows bands at 554 cm^−1^ and 423 cm^−1^, this shift can be related to the deposition of Cu nanoparticle on ZnFe_2_O_4_ [76]. Furthermore, the peak shifts attained due to bending and stretching vibration modes, observed in spectra of ZnFe_2_O_4_-Cu further assured the green synthesis of this nanocomposite.

### 3.3. UV-Vis DRS Study

**Figure 4:**
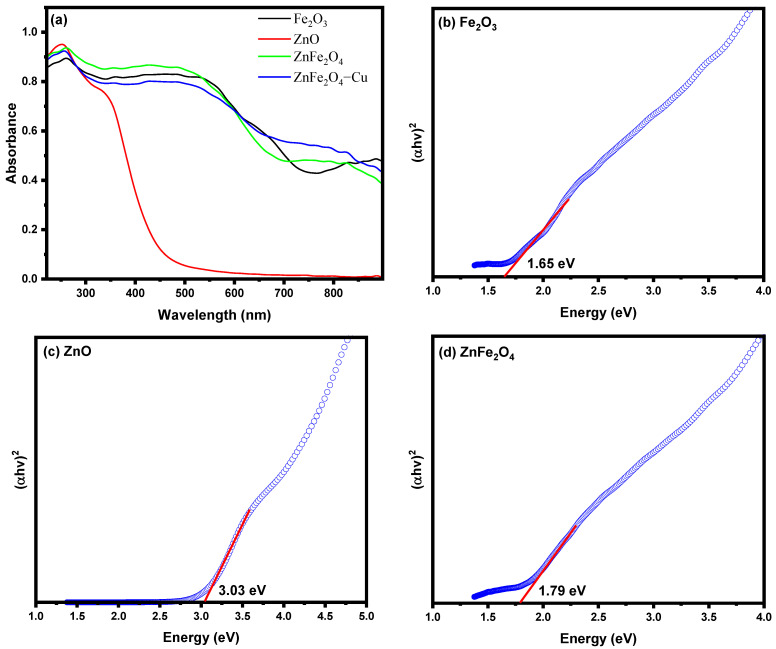

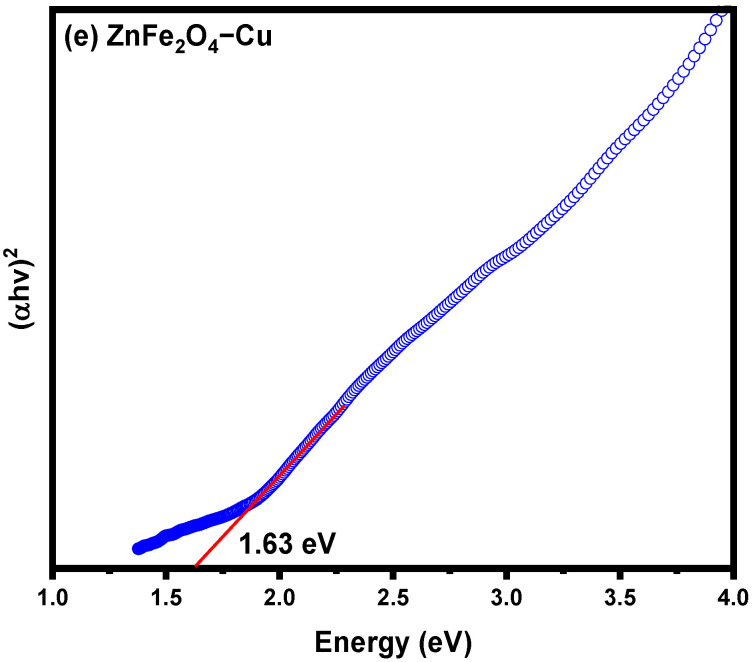
(**a**) UV-Vis diffuse reflectance spectra of Fe_2_O_3_, ZnO, ZnFe_2_O_4_, and ZnFe_2_O_4_-Cu NPs and (**b**–**e**) band gap plots of Fe_2_O_3_, ZnO, ZnFe_2_O_4_, and ZnFe_2_O_4_-Cu NPs.

With the purpose of optimizing light irradiation, a semiconductor with high optical absorbance in the visible range is required for good photocatalytic activity [77]. The optical properties of synthesized nanocomposites have been evaluated using UV-Vis DRS. The UV–Vis spectra of Fe_2_O_3_, ZnO, ZnFe_2_O_4_, and ZnFe_2_O_4_-Cu NPs are illustrated in Figure 4a. Because of the narrow band gap of Fe_2_O_3_, the pure Fe_2_O_3_ particles exhibit high absorption in both the ultraviolet and visible light spectrums. There is an observable light absorption within the wavelength range of 360–555 nm. This absorption is attributed to the electronic transition from the O-2p energy level to the Fe-3d energy level, as shown in references [78,79]. The material ZnO exhibited a broad absorption spectrum ranging from 250 nm to 331 nm. Consistent with expectations, the ZnO nanoparticles exhibited the distinctive spectrum of ZnO, featuring a well-defined fundamental absorption edge that emerged prominently at a wavelength of 331 nm. The UV-Visible spectra of the ZnFe_2_O_4_ sample exhibit significant light absorption within the wavelength range of 355–530 nm, as depicted in Figure 4a. This observation indicates that the heterostructure is responsible for the heightened light absorption characteristic within the UV-visible spectrum. The significant augmentation of light absorption has the potential to impact photocatalytic efficacy through the amplification of photo-generated electrons and holes. When Cu nanoparticles were deposited on ZnFe_2_O_4_, the absorption intensity in the visible region at around 600 nm undergoes a shift towards higher wavelengths (as shown in Figure 4a). This deposition of Cu nanoparticles onto the ZnFe_2_O_4_ heterostructure leads to a significant increase in visible light absorption, particularly at higher wavelengths which can be returned to the charge-transfer transition occurring between the d electrons of the Cu and either the conduction band (CB) or valence band (VB) of ZnFe_2_O_4_ [30]. This finding suggests that the presence of Cu effectively enhances the absorption of visible light. The bandgap energy was estimated using the application of the DRS *Tauc* method, which involves the utilization of the equation (*αhν*)*^γ^ =* A *(hν* − *Eg)*. In this equation, *α* is the absorption coefficient, *h* is the Plank’s constant, *ν* is the frequency of photons, A is a proportionality constant, *γ* equal to 2 ( direct allowed transitions), and *Eg* is a band gap energy [80,81]. The bandgap energy is intercepted by extrapolating the horizontal *y*-axis (photon energy, *hv*) against the *x*-axis, as shown in Figure 4b–e. The direct bandgap energy was determined to be 1.65 eV for Fe_2_O_3_, 3.03 eV for ZnO, 1.79 eV for ZnFe_2_O_4_, and 1.63 eV for ZnFe_4_O_4_-Cu by extrapolating (*αhv*)^2^ against the photon energy (eV), as shown in Figure 4b–e. The results reveal that the bandgap energy for ZnFe_2_O_4_-Cu decreases, which assists the hot electron injection from Cu NPs to the conduction band (CB).

### 3.4. X-ray Photoelectron Spectroscopy (XPS) Analysis

**Figure 5:**
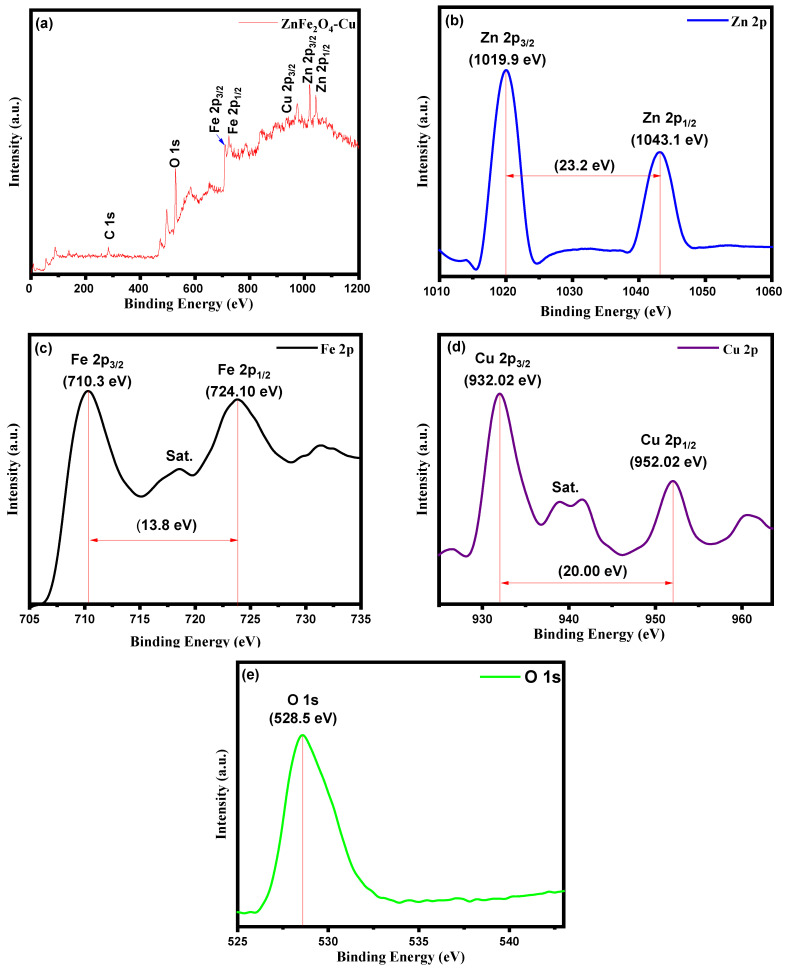
(**a**) Full scan XPS spectrum of ZnFe_2_O_4_-Cu nanocomposites and core level scan of (**b**) Zn 2p, (**c**) Fe 2p, (**d**) Cu 2p, and (**e**) O1s.

The coordination number and various species present in the ZnFe_2_O_4_-Cu nanocomposites analysis using XPS are shown in Figure 5a–e. Figure 5a shows a high-resolution XPS spectrum of the ZnFe_2_O_4_-Cu nanocomposites, which exhibit signals of different elements (Zn, Fe, Cu, and O). The occurrence of the C 1s peak at around 285.6 eV can be returned to the presence of carbon and adsorbed CO_2_ on the surface of the ZnFe_2_O_4_-Cu NPs [82]. Figure 5b–e are the narrow scan XPS spectra for 1s-O, 2p-Fe, 2p-Zn, and 2p-Cu elements, respectively. In the high-resolution Zn 2p spectrum, there are two distinct peaks at 1019.9 and 1043.1 eV, representing the binding energies of Zn 2p_3/2_ and Zn Zn 2p_1/2_, respectively [83]. The XPS spectrum of Fe 2p exhibited two prominent peaks. The peak at approximately 710.3 eV corresponds to Fe 2p_3/2_, while the peak at around 724.10 eV corresponds to Fe 2p_1/2_. These peaks indicate that Fe is predominantly present in a Fe^+3^ [84,85]. The XPS peaks of Cu 2p revealed two prominent peaks at 932.02 eV and 952.02 eV, which are characteristic of reduced copper Cu^0^ phase. Furthermore, the satellite peaks around 938.88 and 941.56 eV can be returned to the presence of Cu^+2^, which can be returned to the incomplete reduction of Cu or may be due to the fact that the Cu can be easily oxidized [86,87]. The peak of O 1s at around 528.5 eV indicates the presence of oxygen in the lattice and oxygen adsorbed on the surface of ZnFe_2_O_4_-Cu NPs [88,89,90]. These results are analogous to earlier reports and confirm the formation of ZnFe_2_O_4_-Cu nanocomposites.

### 3.5. SEM and EDS Analysis

**Figure 6:**
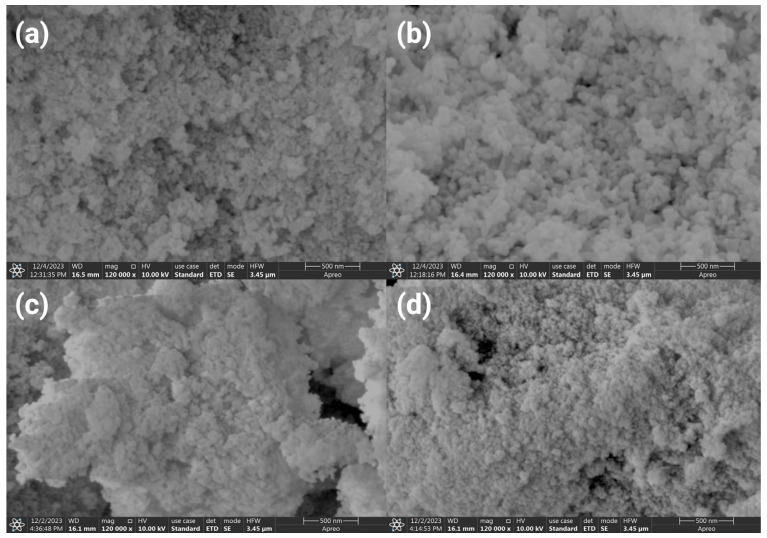
SEM images of (**a**) Fe_2_O_3_, (**b**) ZnO, (**c**) ZnFe_2_O_4_, and (**d**) ZnFe_2_O_4_-Cu NPs.

The surface morphology of the Fe_2_O_3_, ZnO, ZnFe_2_O_4_, and ZnFe_2_O_4_-Cu nanomaterials was analyzed by SEM analysis. Figure 6a–d shows the SEM micrograph of the Fe_2_O_3_, ZnO, ZnFe_2_O_4_, and ZnFe_2_O_4_-Cu nanomaterials prepared by the green synthesis method. SEM images for all samples demonstrate that the particles exhibit a porous structure composed of small particle grains when observed at a magnification scale of 500 nm. In Figure 6a, the irregular-shaped particles of the Fe_2_O_3_ nanoparticles are observed. In addition, surface morphology analysis shows the agglomeration of numerous nanoparticles that can be returned to Van der Waals force and magnetic interactions between the nanoparticles [91]. ZnO nanoparticles, on the other hand, show irregular spherical shapes, as shown in Figure 6b. Moreover, the nanoparticles in the ZnO NPs sample have agglomerated and remain closely packed due to their ultrafine composition. It is clear from Figure 6, images c and d of ZnFe_2_O_4_ and ZnFe_2_O_4_-Cu that after adding the Cu, the agglomeration is decreased. One possible explanation is that pure zinc ferrite exhibits a higher degree of magnetism, leading to significant agglomeration in the SEM image. However, in ZnFe_2_O_4_-Cu, the presence of Cu NPs reduces overall magnetism to a certain extent. This reduction allows for a decreased agglomeration [92].

**Figure 7:**
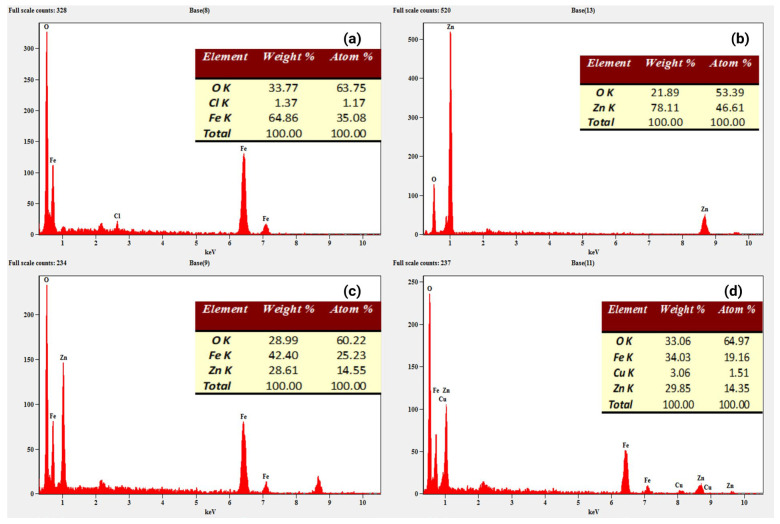
EDX spectra of (**a**) Fe_2_O_3_, (**b**) ZnO, (**c**) ZnFe_2_O_4_, and (**d**) ZnFe_2_O_4_-Cu NPs.

The EDX spectrum of the Fe_3_O_4_ sample in Figure 7a, the prominent peaks corresponding to Fe and O are observed, with a small peak of Cl, possibly indicating a trace amount of chlorides. They can be returned to the metal salt used in the preparation. Similarly, the ZnO sample in Figure 7b exhibits only two distinct peaks attributed to Zn and O. The EDX spectrum of the ZnFe_2_O_4_ nanocomposite sample in Figure 7c reveals Fe, Zn, and O peaks, confirming its successful preparation. Interestingly, a new peak associated with the Cu element is observed in addition to the peaks for Fe, Zn, and O elements in the EDX spectrum in Figure 7d of the ZnFe_2_O_4_-Cu nanocomposites, further confirming the successful preparation of these composite materials. This also confirms the high purity of prepared samples.

To conduct a more in-depth investigation of the EDX output, elemental mapping analysis was carried out for Fe_2_O_3_, ZnO, ZnFe_2_O_4_, and ZnFe_2_O_4_-Cu nanomaterials, as shown in Figure S1a–d. In detail, the images with pink and green dots represent the presence of O and Fe elements in the same region surveyed for Fe_2_O_3_ NPs. On the other hand, the elemental analysis mapping images of EDX of ZnO showed green and yellow spots, which were attributed to O and Zn, respectively. ZnFe_2_O_4_ images exhibited pink, green, and yellow spots, which returned to O, Fe, and Zn elements. These images confirm the homogeneous coexistence of Zn, Fe, and O elements. Moreover, the elemental mapping image of ZnFe_2_O_4_-Cu showed aqua color spots of Cu, uniformly distributed throughout ZnFe_2_O_4_ nanocomposites. This further confirms the successful preparation of ZnFe_2_O_4_-Cu nanocomposites.

### 3.6. TEM Analysis

**Figure 8:**
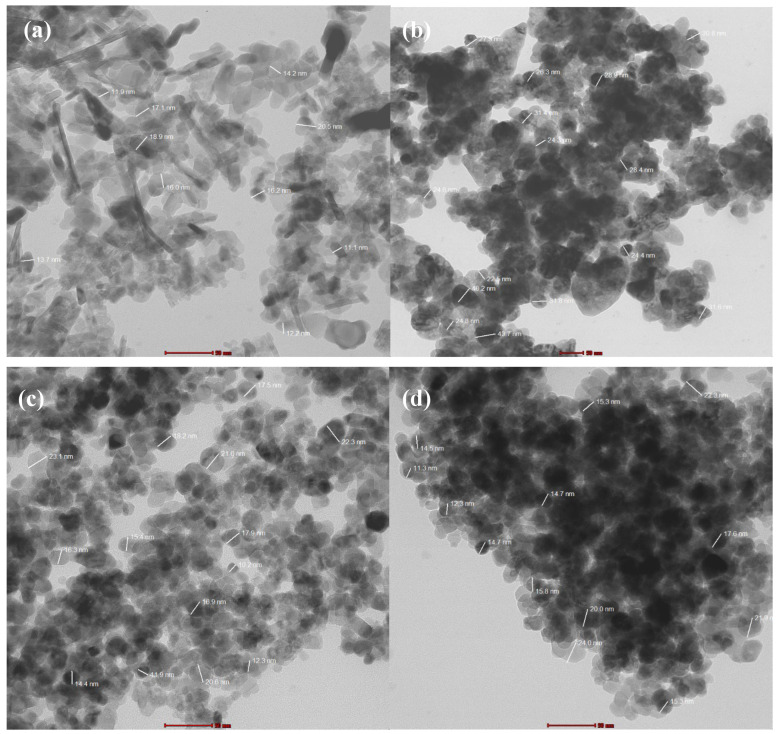
TEM images of (**a**) Fe_2_O_3_, (**b**) ZnO, (**c**) ZnFe_2_O_4_, and (**d**) ZnFe_2_O_4_-Cu NPs.

TEM micrographs for Fe_2_O_3_, ZnO, ZnFe_2_O_4_, And ZnFe_2_O_4_-Cu NPs are shown in Figure 8a–d. The TEM image of Fe_2_O_3_ NPs clearly exhibits spherical, nearly spherical, and rod-like-shaped nanocrystalline phases with an average particle size equal to 15.5 nm, close to the crystallite size obtained by the XRD result. In the case of ZnO NPs, the TEM image demonstrates nearly spherical and hexagonal particle shapes with an average particle size of 29.7 nm. Almost ZnFe_2_O_4_ and ZnFe_2_O_4_-Cu exhibited spherical and nearly spherical nanocomposite shapes. Moreover, careful observation of the TEM image of ZnFe_2_O_4_-Cu nanocomposites unveiled that Cu NPs were deposited on the surface of ZnFe_2_O_4_ in an unsymmetrical manner, where the black spot is Cu NPs while the gray is ZnFe_2_O_4_ nanocomposites. This unsymmetrical deposition led to a long aggregation of ZnFe_2_O_4_-Cu nanocomposites. The average particle size for both ZnFe_2_O_4_ and ZnFe_2_O_4_-Cu nanocomposites were 19.1 and 16.8 nm, respectively, which is slightly bigger than the crystallite size obtained by Scherrer’s formula using XRD data. These results confirm that the ZnFe_2_O_4_ nanocomposites retained their morphology, particularly the high specific surface area. Furthermore, when the samples were calcined at 350 °C for 3 h, the small ZnFe_2_O_4_-Cu nanocomposite particles gradually grew and became more crystalline. This result is also in agreement with that obtained by SEM and BET analyses.

### 3.7. Photoluminescence (PL) Study

**Figure 9:**
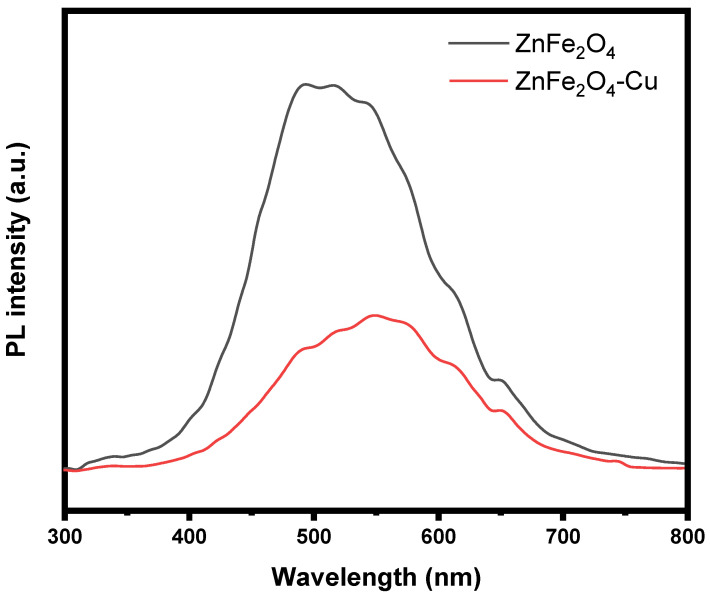
Photoluminescence (PL) spectra of ZnFe_2_O_4_ and ZnFe_2_O_4_-Cu nanocomposites.

The utilization of the photoluminescence (PL) emission spectrum is a prevalent method for the identification of the recombination process of excited charge carriers on a semiconductor material. It is widely recognized that a decrease in the PL signal corresponds to an increased likelihood of separating photogenerated electron-hole (e^−^-h^+^) pairs. This indicates a reduced rate of recombination for the charge carriers, leading to enhanced photocatalytic efficiency. Figure 9 illustrates the PL spectra of both pure ZnFe_2_O_4_ and ZnFe_2_O_4_-Cu nanocomposites. These spectra were obtained by measuring the samples at room temperature using an excitation wavelength of 325 nm. The PL intensity of ZnFe_2_O_4_-Cu nanocomposites exhibits a significant decrease compared to that of pure ZnFe_2_O_4_. This observation suggests that the process of separating photoinduced electron-hole pairs is more effective in ZnFe_2_O_4_-Cu nanocomposites than in pure ZnFe_2_O_4_, owing to the alignment of their band potentials. The observed phenomenon can perhaps be attributed to the creation of vacancies at intermediate energy levels by the introduction of copper doping. Additionally, this phenomenon leads to a decrease in charge carrier recombination.

### 3.8. Nitrogen Gas Physisorption Studies

**Figure 10:**
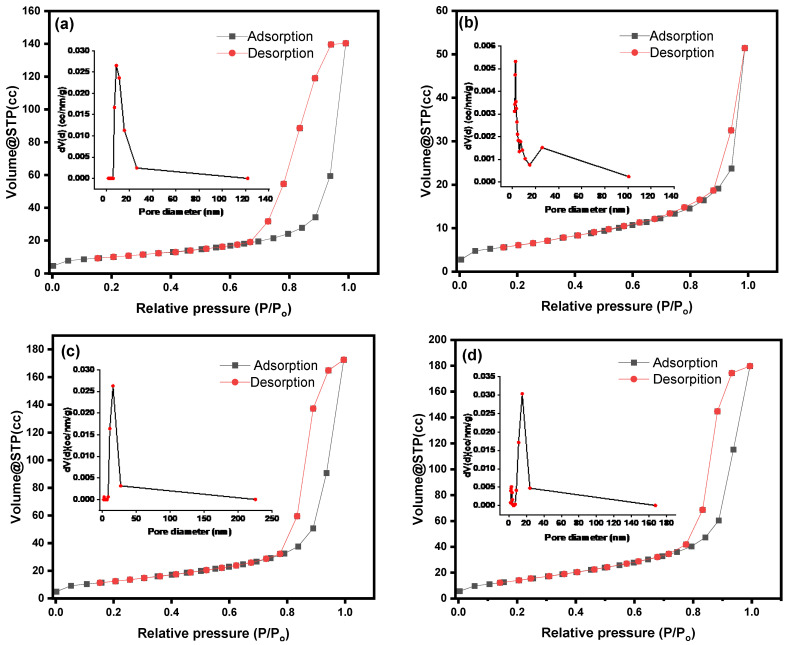
BET adsorption–desorption isotherm of (**a**) Fe_2_O_3_, (**b**) ZnO, (**c**) ZnFe_2_O_4_, and (**d**) ZnFe_2_O_4_-Cu NPs (inset DFT pore size distribution pattern).

The N_2_ physisorption isotherms of Fe_2_O_3_, ZnO, ZnFe_2_O_4_, and ZnFe_2_O_4_-Cu NPs are presented in Figure 10a–d. All samples’ curves showed a typical IV isotherm curve based on the IUPAC classification with an evident hysteresis loop at relative pressure. This confirms the mesoporous properties of as-prepared samples [93]. Figure 10a–d also shows DFT pore size distribution curves, which further demonstrate the formation of mesoporous materials for all prepared samples. Moreover, in all curves, there was a monomodal pore size distribution, one of the most essential characteristics of heterogeneous catalysts. As a result, reactants can reach the active sites more easily. The BET surface area, pore size, and pore volume of the as-prepared samples are given in Table 1. The presence of two different metal ions in ZnFe_2_O_4_ nanoparticles (NPs) in a spinel structure significantly enhanced the surface area, pore size, and pore volume compared to monometallic oxides. Moreover, the surface area of ZnFe_2_O_4_ was increased from 68.814 to 84.866 m^2^/g after doping by Cu, as shown in Table 1. This result is accomplished with the results obtained by SEM and TEM analyses. This can be returned to decrease the agglomeration of particles as a result of reduced magnetic properties of ZnFe_2_O_4_-Cu NPs. Furthermore, the further calcination at 350 °C enhanced the removal of organic compounds and then increased the surface area.

### 3.9. ZnFe_2_O_4_-Cu Nanocomposite as a Photocatalyst for RhB Dye Degradation

The efficiency of ZnFe_2_O_4_-Cu nanocomposites in photodegrading RhB dye molecules was investigated under visible light irradiation. Heteronanocomposites-based metal oxides have been widely used to enhance the photocatalytic degradation of dyes, and these nanocomposites are also employed in other environmental remediations [94,95,96]. The photocatalytic activity of biogenic nanoparticles is generally associated with the recombination of electron-hole pairs in the reaction medium, while other factors such as size, surface area, and radiation source also influence their performance [97,98,99,100].

The presence of heterostructures in ZnFe_2_O_4_-Cu nanocomposites can help to overcome drawbacks associated with photocatalytic performance. For example, ZnO nanoparticles have a relatively high band gap energy, typically around 3.2 eV. This means that they require higher energy photons, typically in the ultraviolet (UV) range, to excite electrons from the valence band to the conduction band and generate electron-hole pairs [101,102]. Additionally, ZnFe_2_O_4_ nanoparticles exhibit high electron-hole recombination rates [103,104].

**Figure 11:**
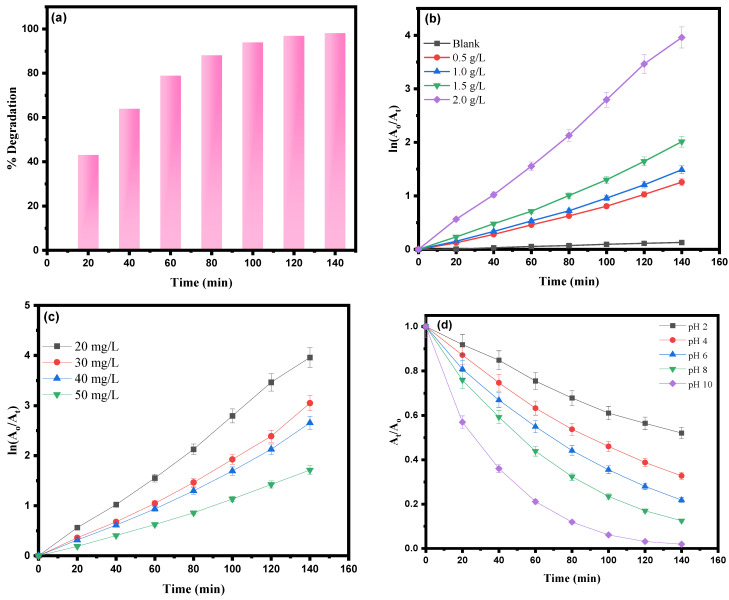
(**a**) Percentage degradation of RhB molecules via ZnFe_2_O_4_-Cu, (**b**) Langmuir–Hinshelwood kinetic model of photocatalytic degradation of RhB dye with various catalyst dosages, (**c**) Langmuir–Hinshelwood kinetic model of photocatalytic degradation of RhB dye with different dye concentrations, and (**d**) effect of pH on RhB dye photodegradation.

Therefore, ZnFe_2_O_4_-Cu nanocomposites can be considered as a novel heterogeneous catalyst under visible light radiation. In this study, a visible light supply using a 300 W Hg lamp with a cutoff filter (420 nm) was employed to generate a continuous light spectrum between 400 and 800 nm for photocatalysis experiments. The photodegradation of RhB molecules was observed through color changes (from pink to colorless) and a continuous decrease in peak intensity at λ_max_ = 554 nm over time. The maximum degradation percentage of RhB dye molecules (98.1%) was achieved after 140 min of exposure, as indicated by the minimum or constant peak intensity. The percentage degradation over time at different intervals was calculated and presented in Figure 11a. The enhanced efficiency of ZnFe_2_O_4_-Cu nano-catalysts for RhB dye degradation can be attributed to the Schottky barrier effect. In ZnFe_2_O_4_ nanoparticles, the recombination of electron-hole pairs occurs rapidly, limiting the photocatalytic efficiency. However, deposition of Cu on the surface of ZnFe_2_O_4_ reduces the recombination of electron-hole pairs and enhances the photocatalytic efficacy [105]. In photolytic reactions with reactive oxidative species (ROS), surface traps increase, and electrons are captured and utilized via Cu-supported ZnFe_2_O_4_ NPs in the degradation of RhB dye molecules [106].

#### 3.9.1. Effect of Photocatalyst Dosage

The photocatalytic properties of ZnFe_2_O_4_-Cu nanocomposites were investigated to degrade Rhodamine B (RhB) dye under visible light irradiation. Different dosages of photocatalysts ranging from 0.5 to 2 g/L were studied in a solution containing 20 mg/L of RhB molecules, and maintained for 140 min. Typically, the photodegradation of dye molecules is detected by observing a color change or the disappearance of color when exposed to visible light. This process involves the conversion of RhB dye molecules into less harmful and biodegradable chemical species, such as superoxide ions (O^2−^), carbon dioxide (CO_2_), or water (H_2_O) molecules [107]. Further, to comprehend the photocatalysis of RhB molecules, we adopted the Hinshelwood kinetic model [108,109]. Accordingly, Figure 11b illustrates the representation of the photodegradation kinetics by plotting ln (A_o_/A_t_) on the *x*-axis and time (min) on the *y*-axis. The experimental results clearly demonstrate that in the absence of ZnFe_2_O_4_-Cu nanocomposites, the degradation of RhB dye was negligible. Furthermore, the catalytic performance improves as the catalyst dose increases from 0.05 to 2.0 g/L. This can be returned to the active sites that were increased as the catalyst dose increased, resulting in higher catalytic efficiency with rate constant k_1_ = 0.02864 min^−1^.

The improved catalytic properties of ZnFe_2_O_4_-Cu nanocomposites can be attributed to their unique characteristics that have been demonstrated through characterization techniques. These characteristics include their optical absorption capability, high surface-to-volume ratio with mesopore size, and the decrease in the recombination between electrons and holes. Attained suitable surface-to-volume ratio, applicable in range small band gap as an effective photo-generated precursor for generation of charge carrier are the prime important factors contributing to photodegradation when ZnFe_2_O_4_-Cu nanocomposites are used as photocatalyst for degradation of RhB dye molecules. Notably, during the investigation, it was observed that a catalytic dose of 2.0 g/L yielded the most favorable results, as shown also in Figure S2a and Table S1. This dosage facilitated enhanced mobility and maximum generation of photo-induced charge carriers, facilitating RhB dye adsorption onto ZnFe_2_O_4_-Cu nanomaterials. Consequently, this led to significantly higher catalytic efficiency in the degradation of RhB molecules.

#### 3.9.2. Effect of RhB Dye Concentration

The relationship between the initial RhB dye concentration and the optimum photocatalytic degradation efficiency was investigated using ZnFe_2_O_4_-Cu nanomaterials as a photocatalyst, employing the Langmuir-Hinshelwood kinetic pseudo-first-order model. A plot of ln (A_o_/A_t_) against time (min) was generated, with a constant catalytic dose of 2.0 g/L and varying dye concentrations ranging from 20 mg/L to 50 mg/L while maintaining a pH of 8 for a 140-min irradiation period, as shown in Figure 11c. Moreover, the experimental data were utilized to calculate the degradation (%) for different RhB dye concentrations at a constant catalytic dose, as shown in Figure S2b. The results revealed that the maximum photodegradation of RhB dye molecules occurred at a concentration of 20 mg/L. However, when maintaining a constant catalytic concentration of 2.0 g/L and varying the RhB concentration within the range of 20 mg/L to 50 mg/L, the degradation efficiency of ZnFe_2_O_4_-Cu nanocomposites gradually decreased. This can be attributed to the fact that at lower dye concentrations, a larger proportion of the available active sites on the catalyst surface are occupied. As a result, there is a higher number of dye molecules interacting with the catalyst, leading to an increase in the degradation efficiency. Conversely, increasing the dye concentration leads to an inhibition effect due to catalytic poisoning and sedimentation. Therefore, this study established that the optimum concentration for RhB dye was 20 mg/L when using a catalytic dose of 2.0 g/L.

#### 3.9.3. Role of pH on RhB Photodegradation

The effect of pH on the photodegradation of RhB molecules utilizing ZnFe_2_O_4_-Cu nanoparticles as catalysts was evaluated in a range from pH 2 to pH 10. As shown in Figure 11d, the obtained data were presented by plotting the A_t_/A_o_ versus the time (min) graph. The experiment was performed in the presence of visible light, with the RhB dye concentration optimized at 20 mg/L and the photocatalyst (ZnFe_2_O_4_-Cu nanocomposite) dose optimized at 2.0 g/L. As a result of the study, a maximum (%) degradation efficiency of RhB molecules was obtained at pH 10, in addition to a minimum degradation of dye at acidic pH 2. This study observed that the effect of pH on the photodegradation of RhB dye molecules via ZnFe_2_O_4_-Cu nanomaterials followed the order of pH 10 > pH 8 > pH 6 > pH 4 > pH 2, respectively. The pH dependence is generally correlated with the surface characteristics of the nanocatalyst. Hence, the efficacy of RhB dye molecule degradation using ZnFe_2_O_4_-Cu is constrained when pH values are altered, which is contingent upon the active surface charge characteristics of these catalysts. The augmentation of pH values leads to an amplification in the net negative charge on the surface of ZnFe_2_O_4_-Cu nanomaterials and then the electrostatic forces of attraction. This is attributed to the adsorption of hydroxide ions (OH^−^) and results in an intensified generation of hydroxyl radicals. On the other hand, at lower pH values, the catalyst surface becomes positively charged, which is unfavorable for this photodegradation reaction [110].

#### 3.9.4. Recyclability Tests

A recyclability test of the synthesized ZnFe_2_O_4_-Cu nanocomposites was performed to evaluate their stability and reusability in the photodegradation of RhB dye. This feature is one of the most critical issues regarding the feasibility of using the heterogeneous catalyst on an industrial scale. The objective was to determine if the ZnFe_2_O_4_-Cu nanocomposites could maintain their catalytic activity over four consecutive cycles under visible light. As a result of the test, it was observed that the photocatalytic degradation of RhB molecules was successfully achieved. Remarkably, complete degradation of the dye molecules was attained within a 140-min exposure time for each cycle, indicating the sustained efficacy of the ZnFe_2_O_4_-Cu nanomaterials as a heterogeneous catalyst.

**Figure 12:**
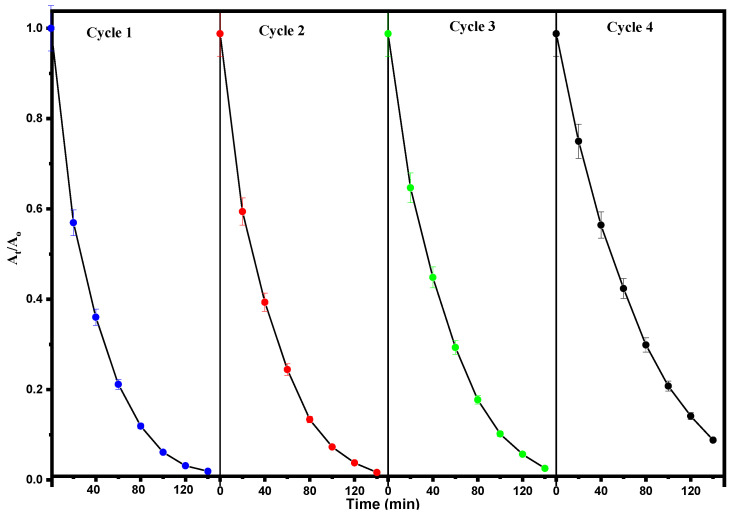
Reusability of photocatalyst after four cycles of reuse.

Figure 12 illustrates how, during four successive cycles, RhB dye molecules were found to degrade by 98–90% in the presence of visible light at total exposure times of 560 min when ZnFe_2_O_4_-Cu nanomaterials were used as nanocatalysts. The results obtained regarding the recyclability of ZnFe_2_O_4_-Cu highlight its potential as a nanocatalyst in the photodegradation process of RhB dye molecules. The negligible decline in the efficiency of ZnFe_2_O_4_-Cu as a nanocatalyst after four consecutive cycles can be attributed to the loss of the ZnFe_2_O_4_-Cu nanomaterial sample during each cycle’s collection and centrifugation procedure.

In general, these results demonstrate that ZnFe_2_O_4_-Cu nanoparticles exhibit recyclability and hold promise as catalysts for the photodegradation of RhB molecules. Additionally, these nanomaterials feature a photocatalytic window that can be effectively employed for the degradation of additional dye molecules. Furthermore, a comparative analysis was conducted by juxtaposing our findings with those of earlier studies, as illustrated in Table 2.

#### 3.9.5. Effect of Addition of Scavengers

**Figure 13:**
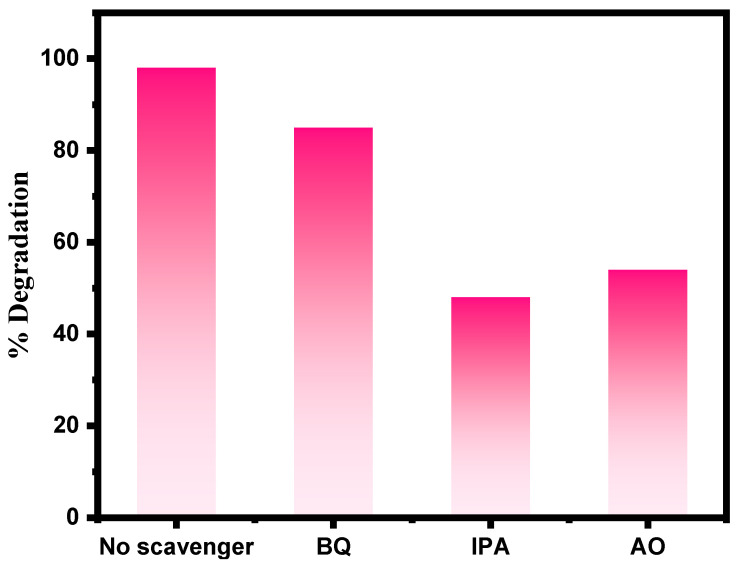
Role of scavengers on photocatalytic degradation of RhB.

In general, reactive oxygen species play an essential role in photocatalysis, including superoxide radical anion ($O_{2}^{-•}$) hydroxyl radical (^•^OH), and holes (h^+^). To evaluate the role of active species in the photodegradation process, suitable scavengers were added to suspensions of RhB dye degradation in the presence of ZnFe_2_O_4_-Cu. Benzoquinone (BQ), isopropyl alcohol (IPA), and ammonium oxalate (AO) were added to capture ($O_{2}^{-•}$), (^•^OH), and (h^+^) respectively, as shown in Figure 13. The RhB dye degradation dropped to 48% and 54% when (IPA) and (AO) were added, whereas the presence of (BQ) resulted in greater RhB dye degradation (85%). Hence, the major species involved in RhB dye degradation by ZnFe_2_O_4_-Cu composite during photodegradation were (^•^OH), followed by (h^+^), and then ($O_{2}^{-•}$).

#### 3.9.6. Mechanism of Photodegradation of RhB by ZnFe_2_O_4_-Cu Nanomaterials

**Figure 14:**
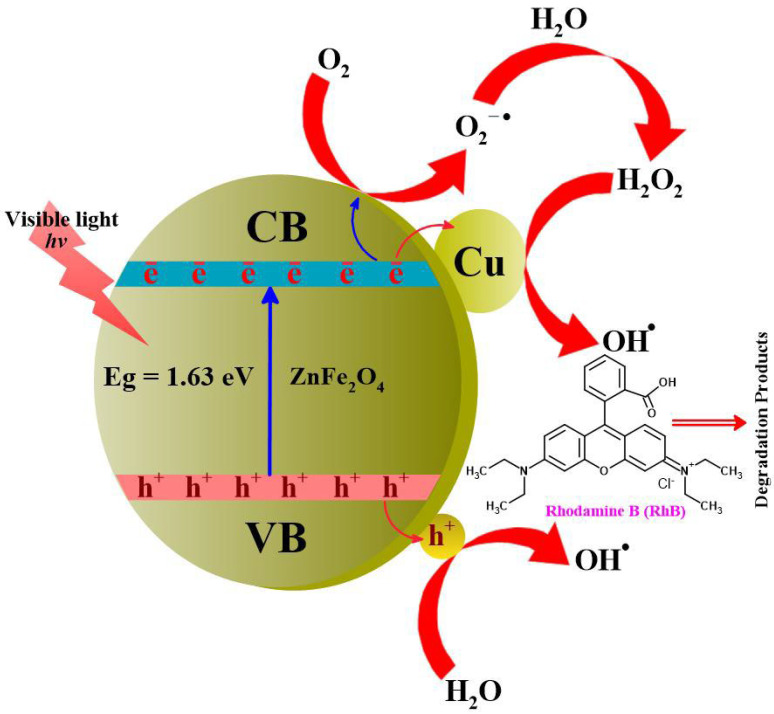
Proposed mechanism of photocatalytic degradation of RhB in presence of ZnFe_2_O_4_-Cu.

Photo-assisted degradation of RhB dye molecules was expected to take place when reactive oxidative species are generated, such as superoxide radical anion ($O_{2}^{-•}$) hydroxyl radical (^•^OH), electron (e^−^), and holes (h^+^) in a photolytic reaction as depicted in Figure 14. Light-induced photocatalysis occurs in the presence of visible light radiations, where electrons on VB of ZnFe_2_O_4_ nanomaterials are excited to CB, leaving behind holes in VB. Excited electrons of CB carry out reduction reactions on the catalyst surface, while holes of VB are responsible for oxidation reactions. Additionally, Cu NPs cause a surface plasmon resonance effect that transfers free electrons to the conduction band of ZnFe_2_O_4_ when their energies exceed the potential of the conduction band. As a result of Schottky energy barriers, hot electrons transferred to ZnFe_2_O_4_ cannot return to Cu NPs. The injection of hot electrons into ZnFe_2_O_4_ causes thermal holes to remain on Cu particles, which causes oxidation [122,123,124,125]. The RhB dye photodegradation involves both reduction and oxidation reactions.

Herewith, the possible stepwise reactions involved in the photodegradation mechanism are represented after the photodegradation of RhB molecules via ZnFe_2_O_4_-Cu nanocomposites. As a result, the Cu-supported semiconductor photocatalyst (ZnFe_2_O_4_-Cu nanomaterials) is expected to be excited by visible light at a lower energy than zinc ferrite’s bandgap. Excitation occurs when electrons excite from VB to CB, leaving a h^+^ in VB. During this study, the nano-catalyst (ZnFe_2_O_4_-Cu nanomaterials) generated the e^−^ in their CB and the h^+^ in their VB of ZnFe_2_O_4_, as a result of visible light irradiation, as per Equation (4). After that, the as-generated e^-^ in CB was reacted with oxygen molecule forms ($O_{2}^{-•}$), followed by reaction between this photo-generated ($O_{2}^{-•}$) and H_2_O in the mixed reaction medium and is converted into $HO_{2}^{•}$ as per Equations (5) and (7). Furthermore, the leaving behind (h^+^) of VB is also adsorbed onto the water molecules, generating the corresponding (^•^OH), radicals, as illustrated in Equation (6). Overall, the photo-generated reaction oxidative species (ROS) are behind the photodegradation of RhB molecules via ZnFe_2_O_4_-Cu nanomaterials, and as a stepwise manner demonstrates such the photocatalytic reduction process from Equations (4)–(9), respectively.

(4) $${ZnFe}_{2}O_{4}-Cu+hv\to {ZnFe}_{2}O_{4}-Cu(e_{CB}^{-}+h_{VB}^{+})$$

(5) $${ZnFe}_{2}O_{4}-Cu{(e}_{CB}^{-})+O_{2}\to {ZnFe}_{2}O_{4}-Cu+O_{2}^{-•}$$

(6) $${ZnFe}_{2}O_{4}-Cu\left(h_{VB}^{+}\right)+H_{2}O\to {ZnFe}_{2}O_{4}-Cu+{OH}^{•}$$

(7) $$O_{2}^{-•}+H^{+}\to HO_{2}^{•}$$

(8) $${OH}^{•},O_{2}^{-•},HO_{2}^{•}+RhB\to {RhB}^{•+}+H_{2}O$$

(9) $${OH}^{•},O_{2}^{-•},HO_{2}^{•}+{RhB}^{•+}\to RhB\;deriv.+\;H_{2}O+CO_{2}$$

## 4. Conclusions

In conclusion, Fe_2_O_3_, ZnO, and ZnFe_2_O_4_ nanocomposites were prepared successfully by the green co-precipitation synthesis method using *Punica granatum* flower extract, followed by Cu deposition on the surface of ZnFe_2_O_4_, which exhibited excellent photocatalysis activity. The *Punica granatum* flower extract contains polyphenol compounds acting as reducing and capping/stabilizing agents during nanomaterials synthesis. Different techniques were used to investigate the physicochemical properties of as-prepared samples. It was found that the deposition of Cu enhanced the absorption of visible light, decreased the (e^−^) and (h^+^) recombination, and increased the surface area compared to ZnFe_2_O_4_. This emphasizes the applicability of using ZnFe_2_O_4_-Cu nanocomposite as a photocatalyst in the degradation of dye molecules. ZnFe_2_O_4_-Cu nanocomposite exhibited high photocatalytic activity, which can be returned to the existence of Schottky energy barriers in ZnFe_2_O_4_-Cu, which inhibited photocarrier recombination. In addition, the increased surface area and mesopore size facilitated the accessibility of RhB dye molecules to the active sites of the photocatalyst. Different parameters, including irradiation time, catalyst mass, dye concentration, and pH, were investigated to determine the optimal conditions for the degradation of RhB dye using ZnFe_2_O_4_-Cu nanocomposite. It was found that under optimal conditions, 98% of the RhB dye was degraded within 140 min. These optimal conditions were achieved with a photocatalyst mass of 2.0 g/L, a RhB dye concentration of 20 mg/L, and a pH of 10. Moreover, the catalyst was used for four consecutive cycles without any significant loss in its performance. The effect of reactive species in this reaction was studied, and it was found that the most effective species in the degradation processes were (^•^OH), followed by (h^+^). Overall, the data support ZnFe_2_O_4_-Cu nanocomposites as potential dye photodegradation targets.

## Figures and Tables

**Table 1:** BET surface area, pore diameter, and pore volumes of Fe_2_O_3_, ZnO, ZnFe_2_O_4_, and ZnFe_2_O_4_-Cu NPs.

Catalyst	S_BET_ (m^2^/g)	Pore Diameter (nm)	Pore Volume (cc/g)
Fe_2_O_3_	35.277	8.764	0.252
ZnO	25.166	2.783	0.081
ZnFe_2_O_4_	68.814	15.821	0.281
ZnFe_2_O_4_-Cu	84.866	15.228	0.294

**Table 2:** Literature survey concerned ZnFe_2_O_4_-Cu nanomaterials in photodegradation of organic dyes.

Photocatalyst	Dye	Light Source	Degradation (%) or Rate Constant of the Reaction (min^−1^)	Ref.
ZnFe_2_O_4_	Malachite green	Visible light	0.96 (min^−1^)	[111]
	Rhodamine B		0.31 (min^−1^)	
Fe_3_O_4_/CuO	Rhodamine B	H_2_O_2_/Visible light	98.9% within 60 min	[112]
ZnFe_2_O_4_	Crystal violet	Sunlight	1296 (min^−1^)	[113]
CuO-ZnO	Methylene Blue	NaBH_4_/Visible light	0.017 min^−1^	[114]
ZnO			0.0027 min^−1^	
ZnO/CuO/ZnFe_2_O_4_	Methyl orange	H_2_O_2_/Visible light	67.8% within 360 min	[115]
Fe_3_O_4_/ZnO/CuO	Methylene Blue	Visible light	0.015 min^−1^	[116]
		UV light	0.009 min^−1^	
Fe_3_O_4_:CuO:5ZnO	Methylene Blue	UV light	0.0068 min^−1^	[117]
ZnO/Cu_2%_ NPs	Rhodamine B	Visible light	90.0% within 100 min	[118]
CuO/Fe_2_O_3_/ZnO	Bisphenol A	Visible light	0.0227 min^−1^	[119]
CuO/ZnO	Methylene Blue	Visible light	98.5% within 150 min	[120]
Cu-ZnO	Methylene Blue	UV light	94% within 120 min	[121]
ZnFe_2_O_4_−Ag	Rhodamine B	H_2_O_2_/Visible light	0.005 min^−1^	[62]
ZnFe_2_O_4_-Cu	Rhodamine B	Visible light	98% within 140 min 0.02864 min^−1^	This work

## Data Availability

The data can be found in both the article and the Supplementary Materials.

## Supplementary Materials

The following supporting information can be downloaded at https://www.mdpi.com/article/10.3390/toxics12010077/s1, Figure S1: SEM-EDX elemental mapping analysis of (a) Fe_2_O_3_, (b) ZnO, (c) ZnFe_2_O_4_, and (d) ZnFe_2_O_4_-Cu NPs. Figure S2: (a) Photocatalytic degradation rate (%) of RhB dye with various catalyst dosages. (b) Photocatalytic degradation rate (%) of RhB dye with different dye concentrations. Table S1: Langmuir–Hinshelwood kinetic rate constant of different parameters for RhB dye degradation.
