# Supplementary material for: Biogenic Punica granatum Flower Extract Assisted ZnFe2O4 and ZnFe2O4-Cu Composites for Excellent Photocatalytic Degradation of RhB Dye
Source: Toxics. 2024 Jan 16;12(1):77. doi: 10.3390/toxics12010077 (PMC10821476; doi:10.3390/toxics12010077)
Supplement: Supplementary file 1 [file toxics-12-00077-s001.zip › toxics-2744501-supplementary.pdf]

toxics

MDPI

Supplementary Materials: Biogenic *Punica granatum* Flower Extract Assisted $ZnFe_2O_4$ and $ZnFe_2O_4$-Cu Composites for Excellent Photocatalytic Degradation of RhB Dye

Amal Alshehri, Laila Alharbi and Maqsood Ahmad Malik

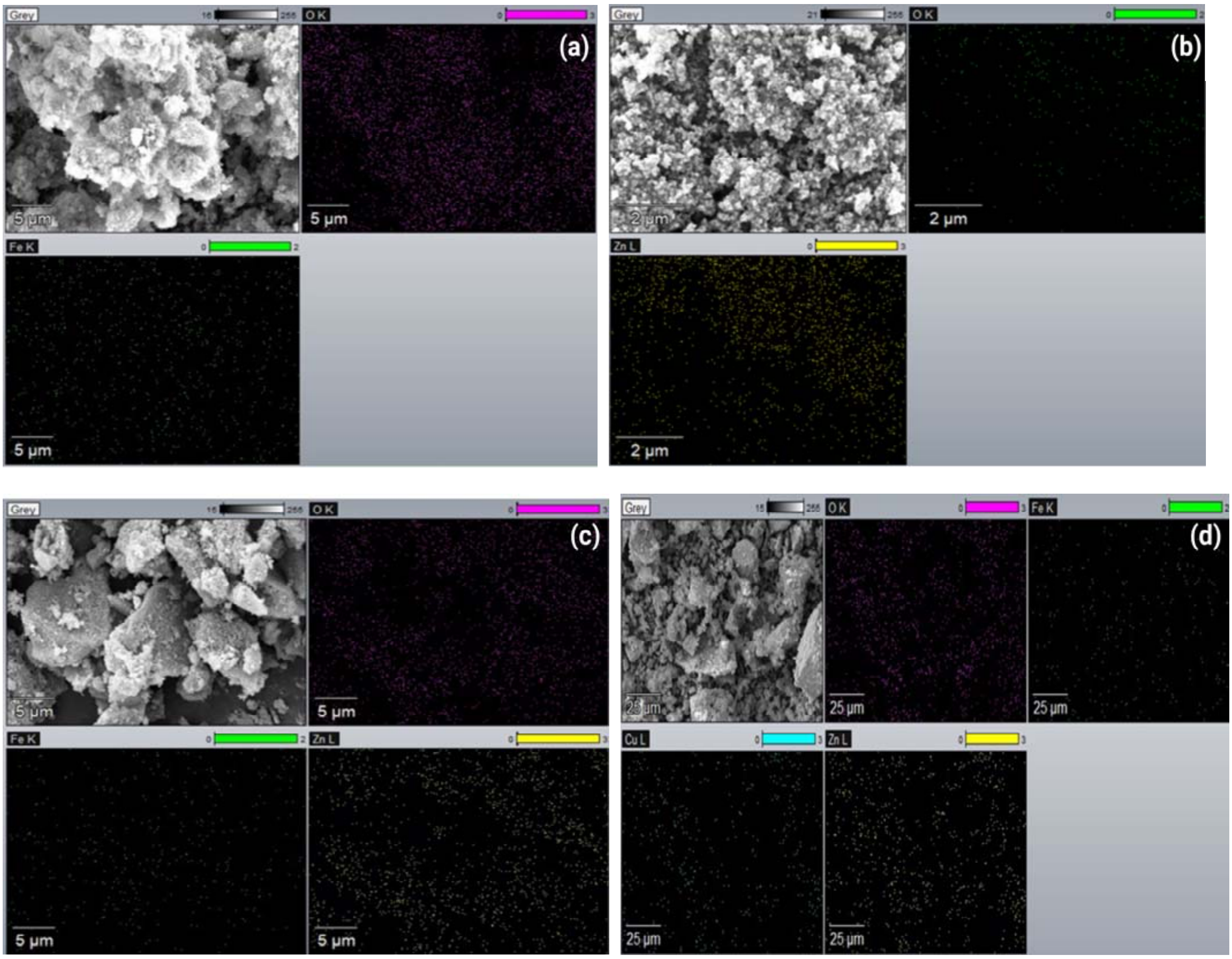

Figure S1. SEM-EDX elemental mapping analysis of (a) Fe_2O_3, (b) ZnO, (c) $ZnFe_2O_4$, and (d) $ZnFe_2O_4$–Cu NPs.

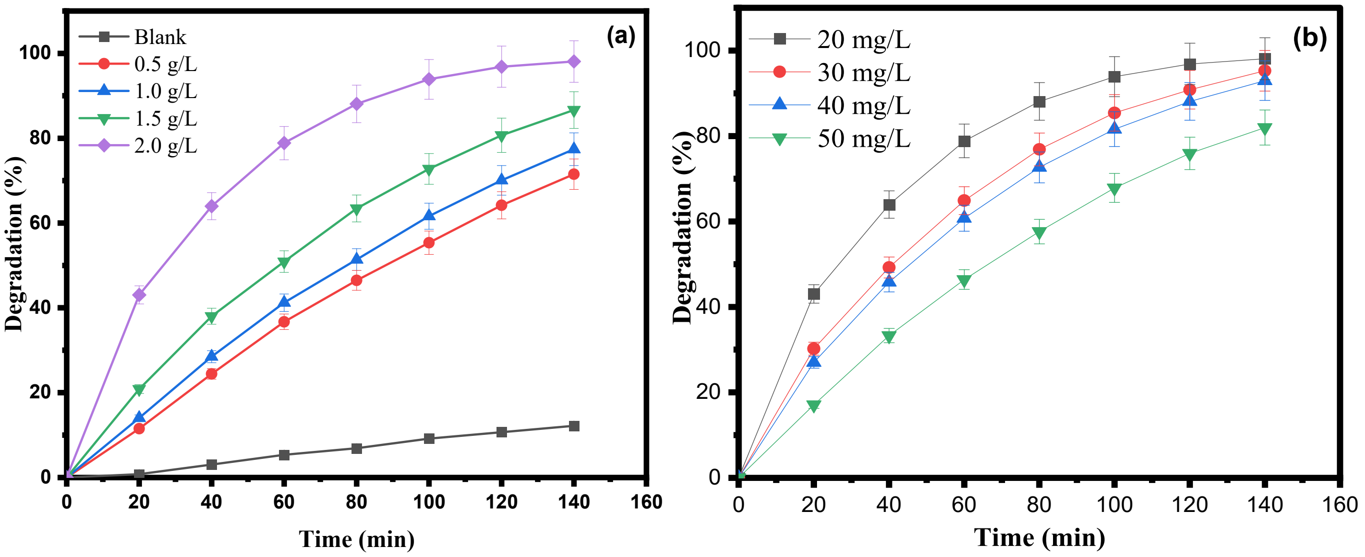

Figure S2. a) Photocatalytic degradation rate (%) of RhB dye with various catalyst dosages. (b) Photocatalytic degradation rate (%) of RhB dye with different dye concentrations.

Table S1. Langmuir–Hinshelwood kinetic rate constant of different parameters for RhB dye degradation.

Parameters		k (min^{-1})
Catalyst dose (g/L)	0.0	0.00098
	0.5	0.00897
	1.0	0.01056
	1.5	0.01423
	2.0	0.02864
Dye concentration (mg/L)	20	0.02864
	30	0.02123
	40	0.01861
	50	0.01227
